# The Worst War of the World

**Published:** 1917-08-11

**Authors:** 


### THE WORST WAR OF THE WORLD.

### II. ITS THIRD ANNIVERSARY IN WESTMINSTER ABBEY.

Throughout the hospital world—of the Allied nations, at any rate—hospital workers who are attendants on the sick have had the horrors of this terrible war brought home to them with a graphic and continuous force, which is not exceeded, if it even be equalled, by that of the fighting men at the Front. Surgeons, Sisters, nurses, orderlies, everybody whose duties keep them in constant contact and association with the wounded, and especially those who have to deal with injuries and the causes of many mutilations, know full well, and feel in their inmost hearts, that there ought to be and can be no peace on earth until our enemies' powers and the causes which spring from them—especially the German militarist system—which has deliberately created, aggravated, and increased with devilish ingenuity, all these terrible outrages upon human beings—have been brought low and rendered impotent for all time. So intense is this conviction, so manifest are the causes from which it springs, that the overwhelming majority of the people of the Allied nations agree that, there can be no peace until victory has placed it in the power of the Allies to ensure, in God's mercy, that never again shall there be a recrudescence of, or the power to attempt to repeat, the horrors, injustices, outrages, and barbarous methods and cruelties perpetrated by the Germans and their allies. The people of the Allied nations, then, at the end of the third year of the war, are as a whole solid in their determination, that, in God's good providence, never again shall it be humanly possible for any repetition of Germany's and her allies' monstrosities to occur. To secure this end, it is absolutely essential that the military power of Germany shall be broken, for otherwise a patched-up peace now must mean "When next?" and not "Never again" after it. In such an event our children and theirs will have to face a new and worse war, when Germany is again ready to attack her neighbours, with a view to their subjection and enslavement. The Allies, then, are bound to fight on until complete victory is theirs, as it must be, under Providence, if we remain steadfast to the end.

### The Spiritual Nature of Man.

On Sunday the 5th inst. the nation was called to give itself to God. The close of the third year of war, found the people, as a whole, anxious to take their part in the Anniversary Services. The spirit of worship and the inclination to prayer have never been stronger, probably, than they are at the commencement of the fourth year of the worst war.

The Archbishop of Canterbury gave the underlying causes in his sermon at the Abbey. He declared: "At this anniversary time we pause and take stock of the three years' outcome. Face it squarely at its grimmest and its saddest; try to belittle nothing, to exaggerate nothing. If we could have foreseen in all their wide ghastliness these three years of human strife and devastation, should we have acted as we did? Would we reverse it now if we could? Ask that question up and down the land, and the answer from almost every thoughtful man and woman would roll back overwhelmingly, 'We were right then, we are right now.' Horrible as it all is, and was, we could do no other. That definite issue of right and wrong, of honour and dishonour, has been no whit impaired."

We have endeavoured during the war to keep in touch with the development and trend of public opinion in this country, and to realise for ourselves exactly what was, and what is, the people's feeling with regard to this war. We believe that practically the whole British nation and Empire are in accord with the force and truth of the statement of the Archbishop of Canterbury just quoted. There can be no doubt that the people of both sexes and in ever-increasing numbers, since this war began, have been quickened to realise more fully and completely than ever before, their spiritual nature, and the need it has for expression and worship. A writer in the *Times* on the 4th inst. forcibly said: "In prayer the soul of man, or the soul of a nation, offers itself as a power, to be possessed and directed by the Divine Mind and Will; what struggles to rise within is the purpose of the Holy Spirit. Prayer at its purest is incarnation. 'It is not ye that speak, but the spirit of your Father that speaketh in you.' Prayer liberates for the Eternal Lord the resources of human personality; in it He acts and speaks, and through those channels, however defective they may be, there flows a Divine desire—there is at work a Divine energy. Prayer is the Spirit of God rising upwards, broken and hindered as yet, but rising out of the human soul in longings and cries which cannot be uttered."

Many who were present in the Abbey on the 5th inst., who took part in the service, must have gained the impression, that, the people present, to a remarkable degree representing the nation, there offered its inner life to be possessed by the Divine Spirit. The service and the worshippers teemed with purified desires, purposes of good for mankind, and uttered vows to serve the Lord God of Holiness. Such a service, and the prayers it involved, can never be in vain, for it has a reality in the spiritual nature of the worshippers, and, the correspondent quoted properly insists, through such "prayer there is opened an illimitable range of power. The nation's own offering, and will, and heart, to the Divine Lord becomes His gift, magnified a thousandfold and given to mankind." "The surrendered will of the nation is added to the sum of powers under the Divine control. There are a myriad approaches to human minds and wills in which He may use that sincerely offered will. The answer to the prayer may come through

Note.—The previous article apppeared on July 28, p. 337.

a flash of insight given to a powerful personality in another land, or through the vision which might awaken repentance, where there is to this hour only blindness of heart, or through some thought of goodwill released in a million hearts at once; or through the moral power added to some faltering and tired statesman. Who can tell what prayers, as units of energy, may become in a world where all things lead out into mystery, and nothing is so unknown as human personality?"

"The nation can give itself to intercession for the world, not greatly concerned to know how its prayers will be answered, but sure that, in so far as the will and conscience and heart are open to the control of the Divine Spirit, the prayer will be true prayer. Amid the mysterious forces at work upon the human scenes, these prayers, since they are of God, will be found again. They will be changed; the nation will never know how its prayers have been answered. But when the records of earth are read in terms of eternal values, it may be seen how the really decisive factors were to be found in the things invisible—in the stirrings and agonies of desire, and groanings which could not be uttered, for these became, through the transforming grace of God, tides of spiritual energy flowing into the dried channels of human life." The whole concern of the worshippers in the Abbey on Sunday last was worship, spiritual outpouring and sustenance, the nation's response to the call to give itself to God.

